# High human cytomegalovirus antigen expression in pediatric medulloblastoma tissue is associated with poor event-free survival

**DOI:** 10.1093/noajnl/vdaf266

**Published:** 2025-12-22

**Authors:** Maria F De la Cerda-Vargas, Jens Schittenhelm, Martin Ebinger, Julian Zipfel, Rudi Beschorner, Ghazaleh Tabatabai, Martin U Schuhmann

**Affiliations:** Department of Neurosurgery and Neurotechnology, Universitätsklinik Tübingen, Tübingen; Department of Neurosurgery and Neurotechnology, Section of Pediatric Neurosurgery, Universitätsklinik Tübingen, Tübingen; Department of Neuropathology, Institute of Pathology and Neuropathology, Universitätsklinik Tübingen, Tübingen; Department of Pediatric Oncology, University Children’s Hospital, Universitätsklinik Tübingen, Tübingen; Department of Neurosurgery and Neurotechnology, Section of Pediatric Neurosurgery, Universitätsklinik Tübingen, Tübingen; Department of Neuropathology, Institute of Pathology and Neuropathology, Universitätsklinik Tübingen, Tübingen; Department of Neurology-Interdisciplinary NeuroOncology, Universitätsklinik Tübingen, Tübingen; Department of Neurosurgery and Neurotechnology, Section of Pediatric Neurosurgery, Universitätsklinik Tübingen, Tübingen

**Keywords:** children, human cytomegalovirus, medulloblastoma, survival

## Abstract

**Background:**

Despite advancements in treatment, 5-year survival for medulloblastoma (MB) remains 60%-70%. Human cytomegalovirus (HCMV) has been implicated as an oncomodulator in MB, but its impact on survival has not been explored. This study evaluates HCMV expression in pediatric MB tissue and its association with molecular risk groups and survival.

**Methods:**

A retrospective study of pediatric MB cases (≤19 years) from 2007 to 2023 was conducted using the WHO 2021 classification, the Northcott brain-tumor classifier, and the SIOP-Europe/ERN PaedCan risk stratification. HCMV immediate-early (HCMV-IE) and late-antigen (HCMV-LA) expression were assessed by optimized immunohistochemistry, and whole-genome sequencing (WGS) data from 20 tumors were analyzed for viral gene expression.

**Results:**

Forty-five patients (mean age 8.2 years; 22% ≤3 years) were included: WNT (18%), SHH TP53-wildtype (18%), and non-WNT/non-SHH (64%). Twenty percent of MB belonged to low-risk, 33% to standard-risk, and 47% to high-risk (HR-MB) groups. Four tumors exhibited MYC/N amplification. HCMV-LA positivity was found in 84% of cases, with 53% showing high expression (≥25% of cells). Cox-regression identified HR-MB (HR = 4.197, *p* = .021) and high HCMV-LA (HR = 4.334, *p* = .027) as independent predictors of poor outcomes. WGS revealed UL88 as the most abundantly expressed HCMV gene (log_2_[TPM+1] ≈ 14-15), with maximal expression in Group 3 MB.

**Conclusions:**

This study provides the first evidence linking high HCMV-LA expression to adverse outcomes and high-risk molecular features in pediatric MB. UL88 emerged as the most strongly expressed HCMV gene across MB samples. These findings suggest a potential prognostic and therapeutic role for HCMV in MB, warranting validation in larger, prospective cohorts.

Key Points

- High-risk MB (HR-MB; HR = 4.197, *p* = .021) and elevated HCMV-LA expression (HR = 4.334, *p* = .027) were independent predictors of poor survival.
- UL88 was the most abundantly expressed HCMV gene, with peak levels in Group 3 tumors.
- HCMV may represent a prognostic biomarker and potential therapeutic target in pediatric MB.

Importance of the Study

This study is the first to assess human cytomegalovirus (HCMV) expression in pediatric medulloblastoma (MB) using the WHO 2021 classification, the Northcott brain-tumor classifier, and the SIOP-Europe/ERN PaedCan risk stratification. HCMV was evaluated with two antibodies (IE and LA) using optimized immunohistochemistry, and inflammatory markers (NF-κBp65, COX2, mPEGS-1, phospho-STAT3) were analyzed across molecular subtypes. Among 45 cases, high-risk MB and elevated HCMV-LA expression (≥25% of cells) were associated with significantly poorer 5-year event-free survival. In high-risk MB, high HCMV-LA retained independent prognostic value beyond clinical risk. Cox regression indicates that high-risk status and elevated HCMV-LA expression are significant risk factors for poorer outcomes. Whole-genome sequencing of 20 MB samples identified UL88 as the most highly expressed HCMV gene, especially in Group 3 tumors. These findings indicate that HCMV expression has independent prognostic significance and may serve as a novel therapeutic target in pediatric medulloblastoma, warranting further validation in larger cohorts.

Medulloblastoma (MB) is the most common malignant brain tumor in children, representing 15%-20% of all pediatric brain tumors across Europe.^1^ In Germany, MB accounts for 18% of pediatric brain tumors.^2^

Despite significant advancements in treatment—including safe tumor resection, chemotherapy (CT), and irradiation—overall survival (OS) rates for pediatric MB remain 60%-70% at 5 years.^3^ Survivors often develop serous long-term effects, including neurological, endocrinological, and psychological complications, in addition to therapy-related immediate acute side effects.^4^

Molecular MB classification into four main groups highlights better prognostic outcomes (5-year OS>90%) in WNT-activated tumors in children (associated with CTNNB1 and APC mutations) in cases without risk features (<16 years, R0, M0, non-LCA, non-*MYC/N-amplified*).^5^ Significantly poorer outcomes are observed in SHH TP53-mutated (SHH-α) cases, which typically occur in patients aged 5-16 years and are associated with a 5-year event-free survival (EFS) of 20%-40%. Among children under 5 years, the SHH-2 subtype is linked to a poor prognosis (5-year EFS of 50%-70%) compared with the SHH-β subtype (infant SHH-1), which is associated with better outcomes (5-year EFS >80%).^5–7^ Notably, non-WNT/non-SHH tumors, especially in Group 3 with *MYC amplification*, have the lowest survival rates in children (OS ∼30% at 5 years). Converserly, Group 4 with *MYCN amplification* and isochromosome 17q [i(17q)] shows better outcomes (5-year OS of 70%-80%).^6^^,^^8^^,^^9^

The *SIOP-Europe/ERN PaedCan consensus*, published in 2024, outlines MB risk groups for patients younger and older than 3 years. SIOP/ERN PaedCan classifies infants under 3 years into three risk groups: *Low -risk* includes TP53-wt tumors without high-risk features (such as MYC/N amplification, large-cell/anaplastic [LCA] histology, residual tumor volume [RTV] >1.5 cm^2^ [R+], metastasis [M+]). *Standard -risk* covers non-WNT tumors (R0/R+) without high-risk features or TP53-wt tumors with M+ (MYC/N non-amplified, DN/MEBN, R0/R+). *High -risk* includes TP53-mutated and/or MYC/N-amplified tumors, non-WNT/non-SHH tumors with M+ (classic histology, R0/R+), and LCA.^10^ Children over 3 years are also divided into three risk groups: *Low -risk* , wich includes WNT tumors (<16 years) or TP53-wt tumors without risk features. *Standard-risk* tumors are WNT (any age, not low-risk) with M+(<16 years) or M0(>16 years), non-LCA, R0/R+, and TP53-wt tumors without risk features (R0/R+), except for Group 4 MYCN-amplified tumors, and non-WNT tumors without high-risk features. *High-risk* tumors include TP53-mutated and/or MYC/N-amplified tumors (excluding Group 4 MYCN-amplified), non-WNT, and WNT tumors (>16 years) with M+, MYC-amplified, or LCA.^10^ The low-risk group has a 5-year OS >90%, while the stndard-risk rates range from 75% to 90%, and high-risk MB tumors have survival rates of 50%-75%.^1^^,^^10^^,^^11^

Given these limitations in treatment and prognosis, there is increasing interest in identifying additional risk markers and novel therapeutic targets for MB. One potential target is human cytomegalovirus (HCMV), a virus involved in the oncomodulation of neuroepithelial tumors, including MB and glioblastoma.^3^^,^^12–14^

HCMV infection occurs in three stages: the immediate-early (IE) stage (3-12 hours postinfection), associated with alpha genes; the intermediate-early stage (12-24 hours), characterized by early antigen (EA) and beta genes; and the late stage (36-72 hours), linked to gamma genes and HCMV-LA expression. During this final stage, HCMV binds to the host DNA in the nucleus, aiding immune system evasion.^15^^,^^16^ After binding to the host DNA, HCMV can alter DNA structure and cause damage,^17^ sequester p53 in the cytoplasm, and inhibit its nuclear function.^18^ Furthermore, HCMV can induce MYC expression ,^19^ dysregulate WNT/β-catenin signaling,^20^ and interfere with SHH pathways^21^^,^^22^ wich are related to MB tumorigenesis.

HCMV also induces a pro-inflammatory tumor microenvironment (TME).^13^ The HCMV-encoded protein US28 activates NF-κB, upregulating COX2,^23^ which leads PGE2 production and drives MB cell proliferation.^24^ COX2 also increases VEGF and Cyclin D1 levels, supporting angiogenesis and cell cycle progression.^23^ HCMV-US28 additionally promotes the proliferation of infected cells by activating the IL-6-STAT3 axis, resulting in higher levels of VEGF and IL-6.^25^^,^^26^ Elevated IL-6 levels futher enhance NF-κB activation driven by the HCMV-US28 protein.^14^^,^^23^

Although the role of HCMV in the TME of MB has been previously described, its impact on survival outcomes remains unclear.^3^^,^^12^^,^^13^ This pilot study investigates HCMV expression (IE and LA antigens) in pediatric MB across all molecular subgroups and risk classifications. Our aim is to explore the relationship between HCMV expression, related TME inflammatory pathway markers, and survival outcomes in a German cohort treated according to *SIOP-Europe/ERN PaedCan guidelines.*

## Methods

### Patient Cohort and Sample Collection

A retrospective study was conducted on tissue microarrays (TMAs) and formalin-fixed paraffin-embedded (FFPE) samples (*n* = 2) from 45 pediatric MB patients (≤18 years) treated at the University Hospital Tübingen, Germany, between 2007 and 2023. TMAs were developed from representative 1 mm central tumor sections, with at least two samples per case, prepared using a standard microarray device, as detailed by Behling et al.^27^ If the tissue was inadequate for TMA, a complete slide analysis was conducted. Thickness: 4-5 μm slides were analyzed using immunohistochemistry (IHC), as described in the following sections. This study was approved by the institutional research board (227/2023BO2). Inclusion criteria considered MB samples classified according to the WHO 2021 criteria,^7^ including the main molecular groups (WNT, SHH, Group 3, and Group 4) according to the “brain tumor classifier” reported by Northcott et al. Heidelberg,^9^ and SIOP-Europe/ERN PaedCan risk groups.^10^ Histological tumor classification and ICH for molecular subgroups were primarily performed at the in-house Institute of Pathology and Neuropathology. WNT tumors were characterized by nuclear and cytoplasmic immunoreactivity for β-catenin and GAB1 negativity. SHH tumors by cytoplasmic positivity for both GAB1 and β-catenin, alongside the determination of TP53 mutation (over 50% nuclear positivity). Non-WNT/non-SHH tumors had to exhibit negativity for beta-catenin, GAB1, and YAP1.^28^ Molecular genetic classification was established at the German pediatric neuropathology reference centers in Bonn and Heidelberg through epigenetic classification using the 450K and 850K Illumina arrays and the Heidelberg Brain Tumor Classifier.^9^  *MYC/N-amplification* was also determined at the Heidelberg Neuropathology Reference Center. All necessary data on molecular classification and clinical follow-up were collected from the hospital information system for retrospective analysis.

### Whole-Genome Sequencing Analysis

Twenty MB samples (21 WGS datasets; EGAD00001000327) representing WNT (*n* = 3), SHH (*n* = 3), Group 3 (*n* = 7), and Group 4 (*n* = 7) were analyzed for expression of Human herpesvirus 5 (HCMV; strain Merlin, NC_006273.2).

### Inflammatory Marker Analysis

Correlations between HCMV expression and inflammatory pathway markers were analyzed using the R2 platform (Medulloblastoma Tumor—Pfister—302—custom—ILMN450 dataset) in 251 pediatric MB cases (age ≤19 years). This dataset comprises gene expression profiles derived from DNA microarray analysis of tumor tissue. Evaluated markers included NF-κBp65 (RELA, NFKB1), COX2 (PTGS2), mPGES-1 (PTGES), and phospho-STAT3 (STAT3).

### Immunochemistry for Cytomegalovirus

**Figure 1.**
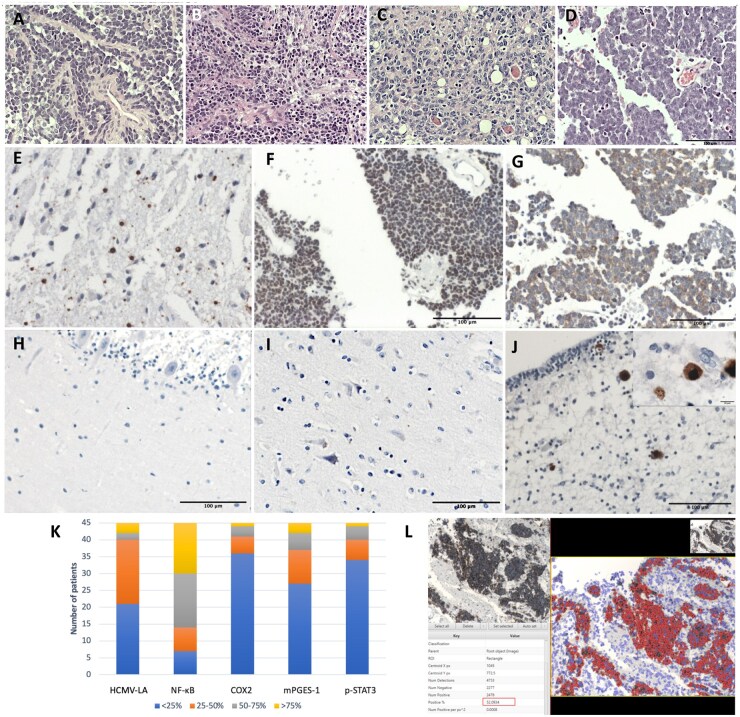
(A-D) Hematoxylin and eosin (H&E) staining showing histological features: (A) classic MB, (B) desmoplastic/nodular, (C) MBNE, and (D) large cell anaplastic. (E) Immunohistochemistry for HCMV late antigen showing low expression (<25% of positive tumoral cells). (F and G) HCMV late antigen IHC, medulloblastoma cases with high expression (≥25% positive tumor cells). (H) Normal cerebellar tissue, (I) Normal brain tissue. (J) Positive control utilizing fetal cerebellum confirmed with meningitis for HCMV, upper quadrant shows an increase of cytomegalic cells (100×), illustrating the contrast with positive cytomegalic cells (∼10 μm) versus positive tumor cells in previous images. (K) Expression of HCMV and an inflammatory marker based on Baraywano’s semi-quantitative scale. (L) Tumor-positive cells quantified with QuPath version 0.5.0 software.

Deparaffinized sections of MB samples were cut into 5 μm slides and were analyzed using an IHC technique for immediate early (IE) and late antigens (LA), using a methodology adapted from Cobbs et al.^29^^,^^30^ Baryawno et al.^12^ and Rahbar et al.^31^^,^^32^ Antigen retrieval was performed using Proteinase 1 for 4 minutes (Prote.1, Ventana) prior to incubation with anti-HCMV antibodies. The Ventana BenchMark Immunohistochemistry System OptiView (Ventana Medical Systems, Tucson, Arizona, United States) was utilized for automated optimized HCMV immunohistochemical staining, involving 60 minutes of primary antibodies incubation at room temperature using mouse monoclonal antibodies (IgG_2a_) against CMV IE antigen (Millipore, MAB81OR, clone 8B1.2, dilution 1:100) and CMV LA antigen (Millipore, MAB8127, clone 1G2.5, dilution 1:100).^30^^,^^33^ Fetal cerebellum diagnosed with HCMV encephalitis; cerebellar and brain samples from healthy children were used as controls (Figure 1).

Samples were assessed under the supervision of a neuropathology specialist (J.S.), based on nuclear and cytoplasmic staining of tumor cells (negativity with ≤1 staining cell).^12^^,^^31^^,^^32^ The samples were categorized according to the semiquantitative scale described for Baraywano et al. <25%, 25%-50%, 50%-75%, and >75% positive tumor cells.^12^ Our series cutoff was based on Rahbar’s threshold in glioblastomas, distinguishing low HCMV expression levels (0 or less than 25% positive tumor cells) from high expression levels (≥25% positive) associated with survival outcomes.^31^^,^^32^ Tumor-positive cells were quantified using QuPath version 0.5.0, a validated system.^34^ QuPath analysis utilized images from representative tumor samples at ×20 magnification, analyzing cell detection, positive cell detection, optical density summation, and nucleus: Eosin OD mean, or Cytoplasm: Eosin OD mean staining (Figure 1).

### Immunochemistry Protocol for Inflammatory Markers

The Ventana BenchMark Immunochemistry System OptiView (Ventana Medical Systems, Tucson, Arizona, United States) was also used for immunohistochemical staining of inflammatory markers in 5 μm slides from TMA or FFPE full slides. Pretreatment was performed with prediluted Cell Conditioning Solution (CC1 or CC2) (pH 6.0) for 32 minutes, followed by incubation with primary antibodies: NF-κBp65 (Cell Signaling, Rabbit monoclonal antibody IgG, dilution 1:800, at 37°C for 32 minutes), phospho-STAT3 (Cell Signaling, clone (Tyr705) (D3A7), dilution 1:400, at 37°C for 32 minutes), COX2 (Biozol, Eching, Germany, mouse monoclonal antibody IgG1, dilution 1:800, at room temperature for 32 minutes), and mPEGS-1 (Cayman Chemical, rabbit polyclonal antibody, dilution 1:200, at room temperature for 32 minutes). Cerebellar tissue from healthy children, colorectal carcinoma metastases, and urothelial cancer was used as controls (Supplementary Material, Figure S1).

Tumor cells were evaluated for nuclear and cytoplasmic staining, with negative cases defined as (≤1 staining cell). We utilized a semiquantitative scale for assessing tumors: <25%, 25%-50%, 50%-75%, and >75% positive tumor cells. Low expression was considered if 0 to <25% of positive cells were found as opposed to high expression (≥ 25% positive tumor cells).

### Extent of Tumoral Resection

The extent of resection (EOR) was classified as follows: complete or near complete tumor removal (R0) confirmed on postoperative MRI with no or residual RTV <1.5 cm^2^, and *subtotal resection* (R+), with RTV >1.5 cm^2^.^11^^,^^35^

### MB Risk Stratification

Risk stratification was performed based on the guidelines outlined above according to the *SIOP-Europe/ERN PaedCan consensus* (2024). This assessment considered high-risk features (MYC/N-amplified, LCA, RTV >1.5 cm^2^ [R+], and metastasis [M+]) across age groups (pediatric cases under 3 years and over 3 years).^10^

### Survival

Follow-up intervals were measured and described in months from the first surgery to the initial or last adverse event, the final oncology follow-up, or death. EFS during follow-up was defined as the period from the date of the first surgery until the first or last significant adverse clinical or radiological event, such as failure to achieve remission (persistence of residual tumor or progression) or a new recurrence, regardless of whether it required additional surgery or death during remission. EFS over a 5-year follow-up period was considered from the date of the first surgery until the date of the first adverse event. We also evaluated EFS during the entire follow-up period, considering the date of the last adverse clinical event in cases with either a single or recurrent adverse events. For this analysis, EFS was assessed using the completed follow-up period, with the date of the most recent adverse event (including primary and recurrence events) as the endpoint, in contrast to the 5-year EFS, which considers only the first secondary event. Cases without adverse events were censored in the analysis. This endpoint has been validated in studies of solid tumors in pediatric patients to overcome some limitations of an OS endpoint.^36^^,^^37^ OS was defined as the time from the first surgery to death from any cause. Survival outcomes were considered for patients with complete follow-up data, molecular classification, and stratification into a risk group.^37^

### Fourth Ventricle Tumor: Resection Technique

Standard surgical technique involves a suboccipital craniotomy without C1 laminectomy and a “U”- shaped opening of the dura, which eases the approach and allows dural closure without duraplasty and prevents cerebrospinal fluid (CSF) leakage.^38^ A telo-velar approach was used and microsurgical resection supported by ultrasound, CUSA, endoscopy, and intraoperative neuromonitoring to achieve so-called maximum safe resection without resecting into the infiltration zone at the floor of the 4th ventricle or alongside the cerebellar peduncles. Retraction was minimized to prevent prolonged compression of healthy tissue.

### Statistical Analysis

Descriptive statistics summarize the data. The Chi-square test (*χ*^2^) examined the associations between HCMV expression and inflammatory markers, considering molecular subtypes, risk groups, and clinical variables. Survival analysis and risk factors were evaluated using Kaplan–Meier and Cox analyses. Statistical significance was set at *p* < .05. Statistical analyses were performed using IBM SPSS Statistics for macOS, Version 30.0 (IBM Corp., Armonk, NY, United States).

HCMV transcripts were quantified from pediatric MB whole-genome sequencing (WGS) BAM files. The BAM files were converted to FASTQ (samtools), aligned to the HCMV Merlin reference genome (NC_006273.2) using Bowtie2, and subsequently sorted and indexed with samtools. Gene-level counts were derived using a GTF annotation and processed in Python 3 (pysam, pandas) to calculate FPKM and TPM values. Heatmaps and dot plots were generated from these normalized counts to visualize HCMV expression across MB samples and molecular subgroups.

## Results

### Study Cohort

Forty-five pediatric MB cases treated between 2007 and 2023 were included. The average age of the patients was 8.21 years (SD = 5.139), with 10/45 (22%) under 3 years. Classic histology was the most prevalent in 32/45 (71%), followed by desmoplastic/nodular (D/N) in 6/45 (13%), large cell/anaplastic (LCA) in 5/45 (11%), and medulloblastoma with extensive nodularity (MBEN) in 2/45 (4%). Metastatic disease (M1-M3) occurred in 18/45 (40%). Complete resection (R0) was achieved in 36/45 (80%), while subtotal resection (R+) was performed in 9/45 (20%). According to SIOP-Europe/ERN PaedCan risk stratification, 9/45 (20%) were LR-MB, 15/45 (33%) SR-MB, and 21/45 (47%) HR-MB (see Table 1). All patients received risk-adapted adjuvant CT and radiotherapy following German pediatric brain tumor protocols.^1^^,^^10^^,^^11^

**Table 1.** Demographic, clinical data, and immunohistochemistry results for the study cohort (total *n* = 45 MB pediatric cases)

Age	*n* (%)
≤3 years old	10 (22)
>3 years old	35 (78)
**Gender**	
Male	25 (56)
Female	20 (44)
**Metastatic disease**	
M0	27 (60)
M1	2 (4)
M2	5 (12)
M3	11 (24)
**Preoperative hydrocephalus**	
yes	29 (64)
no	16 (36)
**Brainstem infiltration**	
yes^a^	17 (38)
no	28 (62)
**Histology**	
Classic	32 (71)
Desmoplastic/nodular	6 (14)
MBEN	2 (4)
Large cell/anaplastic	5 (11)
**Molecular group**	
WNT-activated	8 (18)
SHH-activated and TP53-mut	0 (0)
SHH-activated and TP53-wt	8 (18)
Non-WNT/non-SHH	29 (64)
* Group 3*	12/64 (41)
* Group 4*	17/64 (59)
**MYC/N amplification**	
yes	4 (9)
no	41 (91)
**MB Risk groups^b^**	
LR-MB	9 (20)
SR-MB	15 (33)
HR-MB	21 (47)
**Extend of resection (EOR)**	
RTV no or < 1.5 cm^2^	36 (80)
RTV ≥ 1.5 cm^2^	9 (20)
**HCMV-LA expression levels^c^**	
Low expression	21 (47)
High expression	24 (53)

Abbreviation: MBEN, medulloblastoma with extensive nodularity.

a non-WNT/non-SHH with infiltration in the fourth ventricle.

b LR-MB = low-risk MB, SR-MB = standard risk MB, and HR-MB = high-risk MB (according to SIOP-Europe/ERN PaedCan).

c HCMV-LA expression levels are classified as low (0 or less than 25% positive cells) and high (≥ 25% positive tumor cells).

### Molecular Subgroups and Risk Stratification

**Figure 2.**
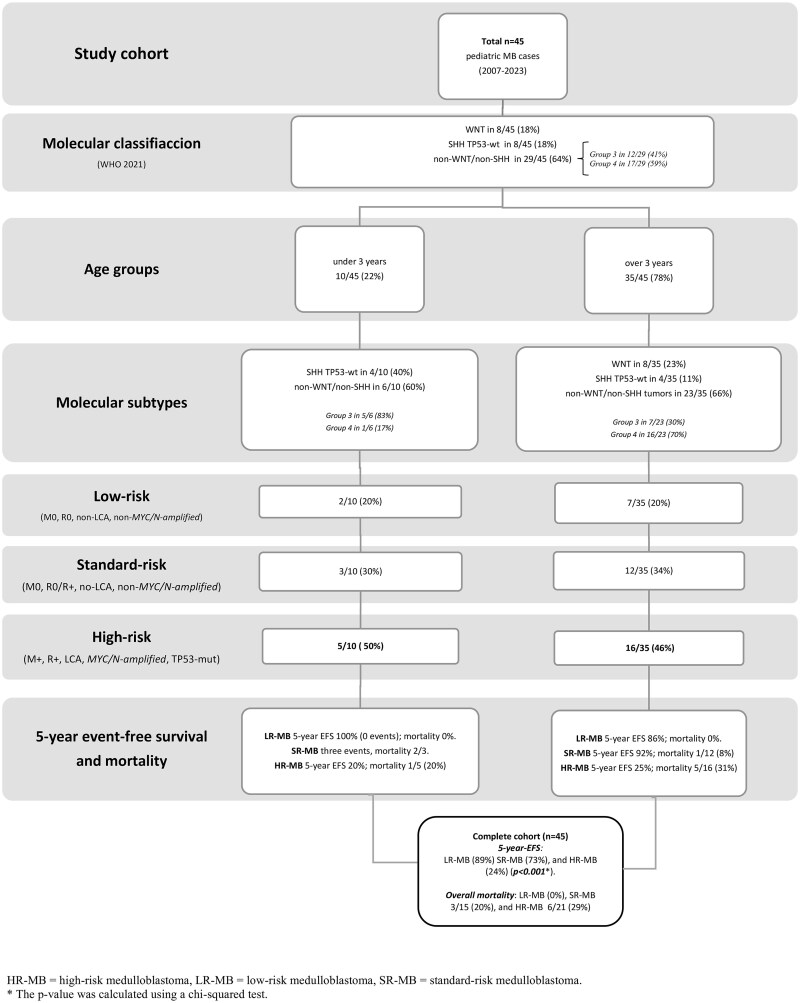
Distribution of medulloblastoma (MB) risk groups according to SIOP-Europe/ERN PaedCan guidelines. *Patients under 3 years* (22%, *n* = 10) included 60% non-WNT/non-SHH and 40% SHH TP53–wild-type cases (non-WNT). High-risk features were observed in 50%, standard risk in 30%, and low risk in 20%. All received adjuvant therapy; CSI was administered in 4 of 10 cases (40%). *Patients aged over 3 years* (78%, *n* = 35) comprised 66% non-WNT/non-SHH, 23% WNT-activated, and 11% SHH TP53–wild-type cases. High-risk features were present in 46%, standard risk in 34%, and low risk in 20%. All received adjuvant therapy, with CSI in 77% (36 Gy in 10 cases, 23.4 Gy in 17 cases). Treatments followed HIT and SIOP-based protocols according to risk stratification.

Eight tumors (18%) were WNT-activated, predominantly low-risk (7/8, 87%), with cytogenetic alterations including monosomy 6 (25%), chromosome 6 loss (37%), combined 6/13q loss (13%), and CTNNB1 mutations (37%). SHH-activated tumors (8/45, 18%) were TP53-wildtype; three were associated with Gorlin-Goltz syndrome, with MYCN amplification in 1/8 (13%). Risk distribution was HR-MB 3/8 (37%), SR-MB 3/8 (37%), and LR-MB 2/8 (25%). Non-WNT/non-SHH tumors comprised 29/45 (64%), with MYC amplification in 3/29 (10%). HR-MB accounted for 18/29 (62%), with 17/29 (59%) exhibiting brainstem involvement. Group 3 tumors (12/29) were evenly split between HR- and SR-MB, with 83% showing brainstem involvement. Group 4 tumors (17/29) had 71% HR-MB and 41% brainstem infiltration. Overall 5-year EFS was 89% (LR-MB), 73% (SR-MB), and 24% (HR-MB; *p* < .001) (see Figure 2).

### HCMV Expression Levels and Inflammatory Markers

**Figure 3.**
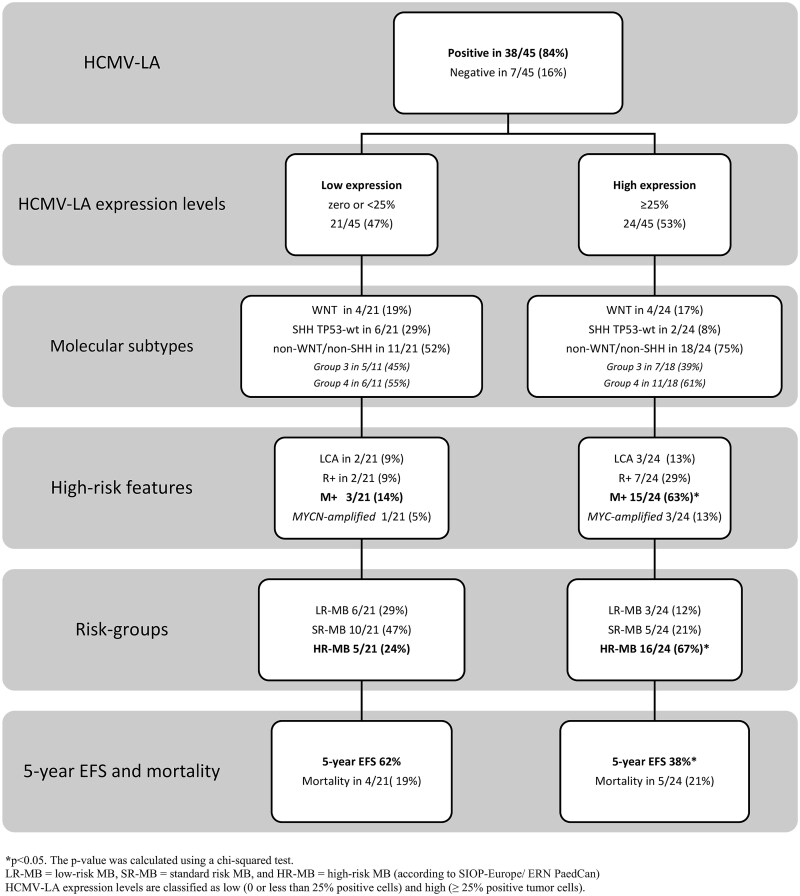
Distribution of the study cohort categorized by HCMV-LA expression levels. The results show that 84% of the samples tested positive for HCMV-LA, with 53% exhibiting high expression (≥25%). High HCMV-LA expression was linked to significantly higher rates of metastatic disease (63% vs 14%, *p* < .001), high-risk medulloblastomas (67% vs 24%, *p* = .016), and lower rates of 5-year event-free survival compared with low-expression cases (38% vs 62%, *p* = .023).

HCMV-LA was detected in 38/45 samples (84%), with 24/45 (53%) showing high expression (≥25% positive tumor cells). Low-expression cases (<25%) comprised 21/45 (47%), according to the cutoff point established in our study based on Rahbar’s previous publications.^31^^,^^32^ Only 3/38 HCMV-LA–positive cases also expressed CMV-IE, all at low levels (Figure 1). High HCMV-LA expression was significantly associated with metastatic disease (15/18, 83%, *p* < .001), HR-MB (16/21, 76%, *p* = .016), and elevated mPGES1 (13/17, 77%, *p* = .015). High HCMV-LA expression correlated with poorer 5-year EFS (38% vs 62%, *p* = .023) (Figure 3). Stratified analyses showed children over 3 years with HR-MB (13/21, 62% vs 38%, *p* = .019) or metastatic disease (13/21, 62% vs 38%, *p* = .001) had significantly higher HCMV-LA expression, and all children under 3 with HR-MB exhibited high HCMV-LA levels (*p* = .038).

**Figure 4.**
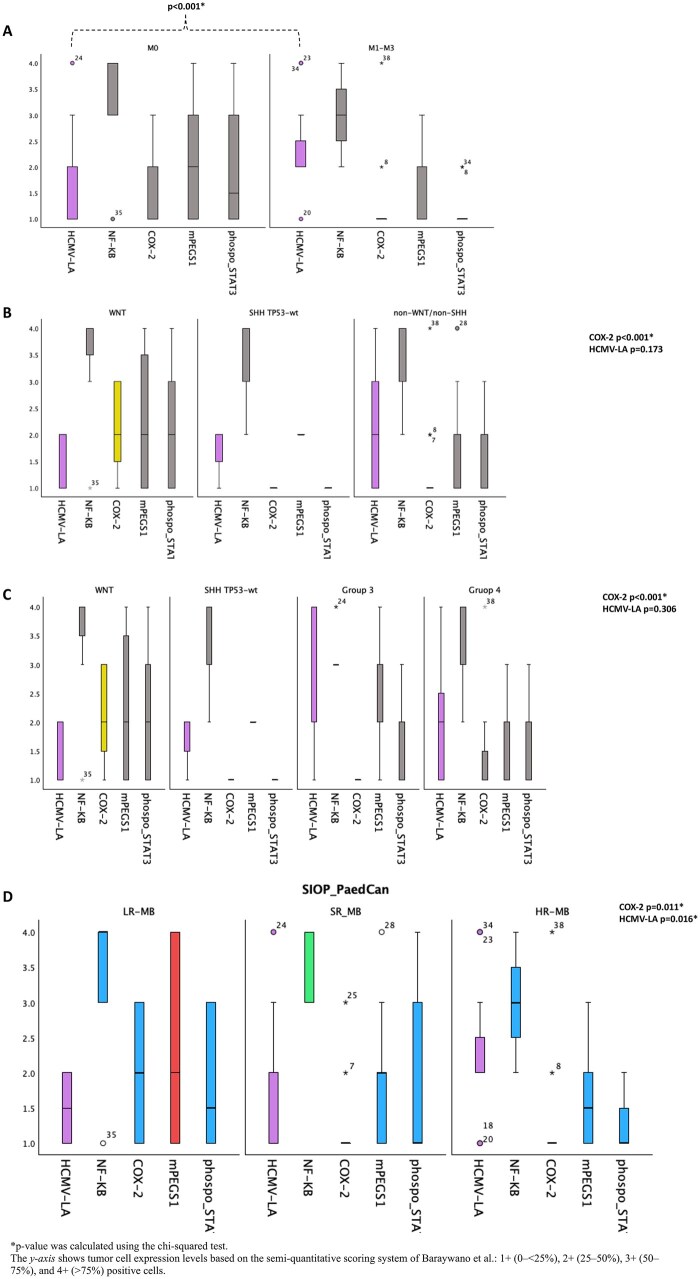
The box-and-whisker plot illustrates the distribution of HCMV-LA and inflammatory markers across different molecular subtypes and risk groups, with a focus on metastasis. The samples were classified using a semiquantitative scale based on *Baraywano et al.* (*y-axis*): 1+, 0% or <25%, 2+, 25%-50%, 3+, 50%-75%, and 4+, >75% positive tumor cells. A cutoff was set to distinguish between low (0 or less than 25%) and high expression (≥ 25%). (A) Patients with metastasis (M+, *n* = 18) showed significantly higher HCMV-LA expression in 15/18 compared with only 3/15 with low expression (83% vs 17%, *p* < .001). (B and C) 6/8 (75%) of WNT tumors exhibited significantly increased COX2 expression compared with 2/8 (25%) with low expression (*p* < .001). Among cases with high HCMV-LA expression levels (*n* = 24), notably 18/24 (75%) were identified as non-WNT/non-SHH tumors (*p* = .173), with 7/18 (39%) in group 3 and 11/18 (61%) in group 4. This finding showed no significant differences when analyzing all molecular subtypes. (D) For risk groups, 16/21 (76%) of high-risk tumors had high HCMV-LA expression compared with 5/21 (24%) with low expression (*p* = .016). Meanwhile, 5/9 (56%) of low-risk tumors showed a significant association with high COX2 expression, compared with 4/9 (44%) with low levels (*p =* .011). The other inflammatory markers did not show significant differences.

Inflammatory marker analysis revealed high expression (≥25% positive tumor cells) in 38/45 (84%) for NF-κBp65, 9/45 (20%) for COX2, 17/45 (38%) for mPGES-1, and 11/45 (24%) for phospho-STAT3. All MB tumors were positive for COX2 (*n* = 45), but most exhibited low expression levels (<25%). Interestingly, COX2 high-expression tumors were exclusively classic histology (9/9), predominantly WNT (6/9, 67%, *p* < .001), and associated with low-risk features (5/9, 56%; *p* = .011) in patients over 3 years (patients under 3 years did not present WNT tumors, and none showed high COX2 expression levels) (Figure 4).

The other inflammatory markers (NF-κBp65, mPEGS-1, and phospho-STAT3) did not show significant differences in expression, age group, molecular group, and risk stratification.

### Clinical Outcomes

Among 45 patients, 21 (47%) experienced ≥1 adverse event, with 15 presenting a single event and 6 recurrent events. The remaining 24 (53%) were event-free, yielding a 5-year EFS of 53%. Mean follow-up was 53 months (range 0-169). Early adverse events were significantly associated with metastasis (78% vs 22%, *p* < .001), preoperative hydrocephalus (62% vs 38%, *p* = .005), high HCMV-LA expression (62% vs 38%, *p* = .023), and high-risk MB (76% vs 24%, *p* < .001). Age stratification showed similar trends in HR-MB patients under and over 3 years (80% vs 75%, *p* < .001).

### Overall Survival

Overall mortality was 20% (9/45), predominantly in metastasis (56%), HR-MB (67%), and non-WNT/non-SHH tumors (89%). MYC/N-amplified cases (4/45) had significantly poorer OS (median 13 months, *p* = .013) (see Table S1 in the Supplementary Material).

### 5-Year EFS

**Figure 5.**
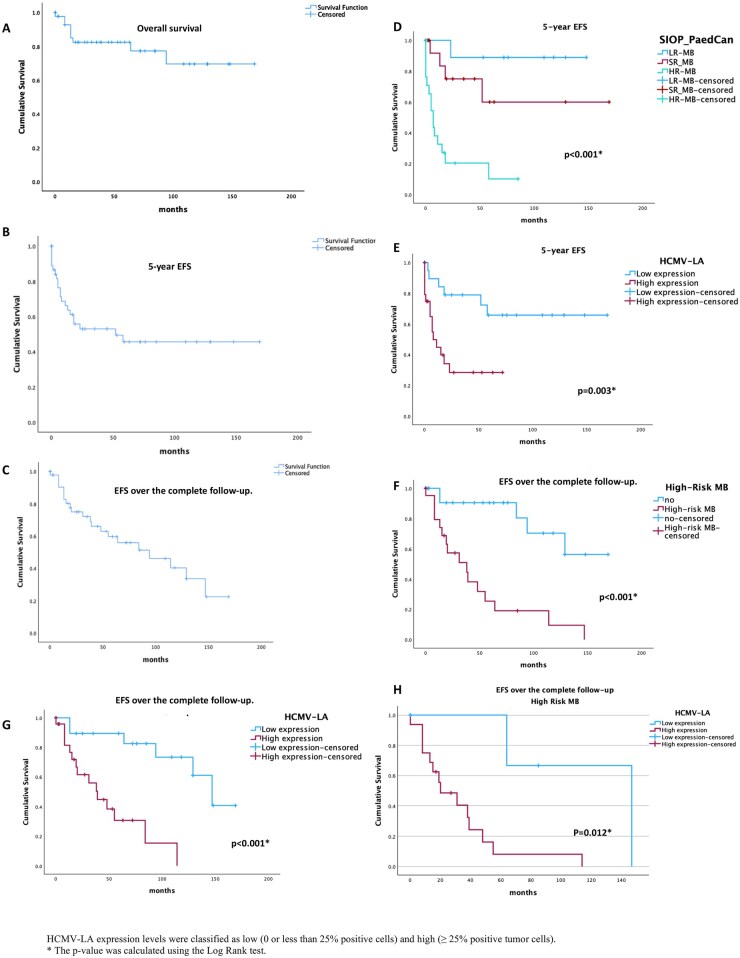
Kaplan–Meier analysis. (A) Overall survival (OS) in the study cohort, considering only deaths; 9 out of 45 (20%) were events. The average OS was 129 months (SD = 11, 95% CI 107–152 months). (B) The 5-year event-free survival (EFS) analysis includes the date of the first adverse event, which occurred in 21 out of 45 patients (47%), with an average of 84 months (SD = 12, 95% CI 59–109 months) and a median of 52 months. (C) EFS was assessed over the entire follow-up period, using the most recent adverse event date as the endpoint. 21 out of 45 patients (47%) experienced adverse events, including 15 with only one event and 6 with recurrent events. The median time to the last recorded event was 90 months (SD = 10), with a median of 94 months. (D) The 5-year EFS considering risk medulloblastoma (MB) stratification indicates that patients with high-risk features showed poorer EFS (median: 7 months) compared to other risk groups (*p* < .001). (E) In the 5-year EFS analysis based on HCMV-LA expression levels, cases with high expression had poorer EFS (median: 8 months) than those with low expression (mean: 120 months; *p* = .003). (F) High-risk MB cases exhibited poorer outcomes (median: 38 months) compared with other risk groups (mean: 32 months, *p* < .001). (G) MB cases with high HCMV expression levels (≥ 25% positive tumor cells) presented significantly poorer outcomes than low-expression cases (median 39 vs 147 months, *p* < .001). (H) Stratified analysis identified that high-risk MB cases (*n* = 21) with HCMV-LA expression (16/21) showed significantly poorer outcomes (median 20 vs 147 months, *p* = .012) compared to low-expression cases.

5-year EFS was significantly shorter in patients with subtotal resection, metastasis, preoperative hydrocephalus, MYC/N amplification (*p* < .005), particularly HR-MB (median = 7 months, *p* < .001, Figure 5D), and high HCMV-LA expression (median = 8 months, *p* = .003, Figure 5E) (see Supplementary Table S1 for full data).

A stratified analysis of non-WNT/non-SHH tumors showed that high HCMV-LA expression was linked to significantly worse EFS (median 8 months) than low expression (*p* = .021). This was especially true in Group 3, where 5/7 patients with high HCMV-LA had events and shorter median EFS than Group 4 (7/11 events; median 7 vs 11 months; *p* = .025). Inflammatory markers showed no significant differences in expression or time to adverse events.

### EFS Over the Complete Follow-Up

Over the entire follow-up, 21 out of 45 patients (47%) experienced adverse events, including 6 with recurrent events. The median time to the last recorded event was 94 months. High-risk MB patients had significantly shorter EFS (median: 38 months) compared with standard-risk (129 months) and low-risk MB (135 months, *p* < .001, Figure 5F). Similarly, high HCMV-LA expression was linked to poorer outcomes (median: 39 vs 147 months for low expression, *p* < .001, Figure 5G). A stratified analysis, it was identified that cases with high-risk MB and high HCMV-LA expression experienced significantly poorer outcomes compared to low-expression cases (median 20 vs 147 months, *p* = .012, Figure 5H), demonstrating that HCMV-LA provides independent prognostic information beyond MB risk status. Furthermore, non-WNT/non-SHH tumors, particularly Group 3 with high HCMV-LA, also exhibited significantly shorter EFS (median: 8 vs 38 months for Group 4, *p* = .003). Additional factors associated with poorer EFS included subtotal resection, metastasis, preoperative hydrocephalus, MYC/N amplification, and high-risk MB (*p* < .005). Inflammatory markers showed no significant effect on time to adverse events (see Table S1 in the Supplementary Material).

### Cox Regression Analysis of EFS

A multivariable Cox proportional hazards model identified age ≤3 years (HR = 3.521, *p* = .012) and HR-MB (HR = 6.594, *p* = .002) as independent adverse prognostic factors for 5-year EFS. Throughout the entire follow-up period, HR-MB (HR = 4.197, *p* = .021) and high HCMV-LA expression (HR = 4.334, *p* = .027) remained independent predictors of poor outcomes (see Table S2 in the Supplementary Material).

### WGS and HCMV Gene Expression

Heatmap analysis of 20 pediatric MB WGS samples revealed distinct HCMV gene expression patterns. UL88, UL60, UL99, and UL76 were consistently highly expressed, with UL88 showing the strongest expression across nearly all tumors (see Figure S2 in the Supplementary Material). Dot plots demonstrated that UL88 expression was highest in Group 3 (TPM ≈ 2,500,000) and Group 4 (TPM ≈ 2,000,000), with lower levels in SHH and WNT subgroups (see Figure S3 in the Supplementary Material).

### Inflammatory Gene Expression in WGS Data

Analysis of inflammatory pathway genes corresponding to IHC markers—NF-κBp65 (RELA, NFKB1), COX2 (PTGS2), mPGES-1 (PTGES), and phospho-STAT3 (STAT3)—using the R2 platform demonstrated subgroup-specific expression patterns. PTGS2 and PTGES were significantly elevated in WNT tumors (PTGS2 median = 0.85, Q25-Q75 = .77-0.88; ANOVA: *F* = 4.015, *p* = .031; PTGES median = 0.86, Q25-Q75 = 0.84-0.89; ANOVA: *F* = 12.276, *p* = 1.63 × 10^−7^), consistent with activation of the COX2/PGE2 pathway (see Figure S4C and D in the Supplementary Material). STAT3 expression was significantly higher in Group 3 and Group 4 tumors (Group 3 median = 0.92, Q25-Q75 = 0.91-0.94; Group 4 median = 0.92, Q25-Q75 = 0.90-0.93; ANOVA: F = 12.187, *p* = 1.83 × 10^−7^). In contrast, RELA and NFKB1 did not show significant differences across molecular subgroups (RELA: *F* = 0.664, *p* = .575; NFKB1: *F* = 1.223, *p* = .302), indicating that NF-κB signaling is not subgroup-specific.

Overall, these findings align with IHC results and highlight a subgroup-specific activation of the COX2/PGE2 inflammatory pathway in WNT tumors, while NF-κB activity remains consistent across MB subgroups. Combined with the HCMV expression data, this suggests a potential interaction between viral gene activity and tumor-associated inflammatory pathways, particularly in high-risk and Group 3 tumors.

## Discussion

HCMV is implicated as a trigger in the inflammatory pathways of the medulloblastoma TME. However, its relationship with survival outcomes remains unclear.^3^^,^^12^^,^^13^ HCMV fulfills all the landmarks of a cancer-oncogen^39^ and has been found to influence survival in other neuroepithelial tumors, such as glioblastoma.^40^ This mono-center pilot study evaluates HCMV IHC expression levels in MB tissues across all molecular subtypes and risk groups and explores their correlation with clinical outcomes in pediatric patients.

Our findings reveal that 38/45 patients (84%) were positive for HCMV late antigen, indicating chronic or latent HCMV in most tumors, as the late antigen typically appears after 72 hours postexposure. This contrasts with previous studies reporting a higher prevalence of HCMV immediate-early antigen (HCMV-IE), a rare finding in our cohort.^3^^,^^12^^,^^13^ We mainly analyzed FFPE TMAs and few FFPE whole slices by an automated, optimized IHC technique with a 60-minute HCMV antibody incubation. This reliable method,^30^^,^^33^ validated with strong HCMV-positive controls, supports the robustness of our results. These findings suggest a latent HCMV presence, rather than an active infectious process in the tumor tissue, which can act as an onco-modulator inducing inflammatory pathways and potentially influencing tumor growth and metastasis.^16^ Although our study did not find a direct correlation between inflammatory markers and survival, the increased expression of these, particularly NF-κB and mPGES1, supports our hypothesis that HCMV functions as an onco-modulator, activating the inflammatory pathway. However, further studies are needed to gain a deeper understanding of this mechanism.

Few studies have explicitly focused on HCMV in pediatric MB, with most examining brain tumors in general instead of concentrating specifically on MB. Furthermore, these studies have mainly analyzed HCMV positivity without investigating its relation to clinical outcomes.^3^^,^^12^^,^^13^^,^^17^^,^^41–44^ Differences in methodology, particularly regarding antibody selection, have led to inconsistencies among studies. Notably, two studies that reported negative results utilized the DDG9/CCH2 clone, known to have the lowest sensitivity for detecting HCMV in tumor tissue,^45^ and three of them, which analyzed HCMV genetic material by PCR, yielded a negative result.^42^^,^^43^^,^^46^ PCR has shown the lowest sensitivity in detecting HCMV infection in FFPE tumor tissue in brain tumors.^45^ Furthermore, PCR seems more useful for detecting active HCMV infections by analyzing specific fluids (blood, urine, or saliva), but it has been reported to lack sensitivity for detecting HCMV presence in the CSF of infants with confirmed congenital HCMV infection.^47^^,^^48^ The low sensitivity of PCR in detecting HCMV in FFPE, along with the limitations in identifying HCMV expression in tumor tissue when analyzing specific tumor cells, and the lability of genetic material (RNA/DNA) in FFPE, which can degrade and fragment due to the fixation process with formalin,^43^^,^^45^ explain the disadvantages of this technique for detecting HCMV in brain tumor tissue, where HCMV plays a more latent/oncomodulatory role.^13^ However, active HCMV infections have been reported in MB cases after receiving adjuvant therapy.^49^ There is no consensus on the optimal method for detecting HCMV in MB, and the lack of research on survival outcomes may be a significant reason for the inconclusive results reported in previous studies.^3^^,^^12^^,^^13^^,^^17^^,^^41–44^ Nevertheless, there are numerous studies focusing on the presence and expression of HCMV and outcomes concerning other neuroepithelial brain tumors, specifically glioblastomas.^40^^,^^45^^,^^50^ Based on these studies, we utilized an optimized immunohistochemical technique for two HCMV antigens (IE and LA), using an antigen retrieval protocol, which demonstrated the most sensitive method for detecting HCMV proteins in tumor tissue (84.2%).^12^^,^^45^

The reported overall seroprevalence of HCMV in children in Germany is 27%.^51^ In our study, HCMV-LA positivity was detected in 84% of MB samples, with higher expression in MBs featuring high-risk characteristics. Additionally, HCMV-LA expression and high-risk MB groups were linked to poorer EFS outcomes and were identified as independent risk factors for poor prognosis in the Cox analysis, highlighting the significance of both in our cohort. These findings suggest that HCMV presence may contribute in pediatric MB, because its presence was mainly associated with high-risk tumors. Furthermore, congenital CMV infection can dysregulate the SHH and WNT pathways, which are crucial for the development of the central nervous system (CNS), resulting in abnormal neural differentiation.^22^ Alterations in these pathways have also been associated with two of the current MB molecular subtypes.^5–7^ HCMV LA positivity is associated with the late stage of HCMV infection, when the virus binds to the host DNA in the nucleus, where it can produce DNA damage^17^ and promote immune system evasion, avoiding cell death.^15^^,^^16^ After binding to DNA, HCMV can alter its structure, causing damage,^17^ sequester p53 in the cytoplasm, and inhibit its nuclear function.^18^ Additionally, HCMV can induce the expression of MYC,^19^ downregulate WNT/β-catenin signaling,^20^ and dysregulate SHH pathways^21^^,^^22^ related to MB tumorigenesis and risk MB features. HCMV can sequester p53 in the cytoplasm and inhibit its expression in the nucleus.^18^ This could explain why in a series with high CHMV-LA expression, TP53-mutant tumors were not detected, even in SHH cases that exhibited high-risk features. Furthermore, immunosuppression within the TME may trigger the expression of a latent HCMV infection, potentially contributing to more severe disease and poorer outcomes. HCMV oncomodulation could also be associated with the established molecular mechanisms linked to reduced survival, particularly in high-risk tumors.^10^^,^^11^ Although further research is necessary for understanding the complete influence of HCMV on the medulloblastoma TME.

Analysis of WGS data from 20 pediatric MB tumors revealed consistent HCMV gene expression, with UL88 showing the strongest signal, especially in high-risk Group 3 tumors. Inflammatory gene analysis confirmed subgroup-specific patterns, with elevated COX2 (PTGS2) and mPGES-1 (PTGES) expression in WNT tumors, while NF-κB signaling remained stable across subgroups, consistent with IHC results. These findings suggest a potential interaction between viral activity and tumor-associated inflammation. Supporting prior research, Baryawno et al.^12^ reported that 92% of primary MBs expressed immediate-early (IE) HCMV proteins and 73% expressed late proteins, including UL83. More recently, Kumar et al.^52^ identified UL88 as a novel antagonist of innate immunity, enhancing HCMV spread via MyD88. Collectively, these results underscore the potential role of UL88 in modulating immune responses within the MB microenvironment and its contribution to oncogenic signaling, supporting the hypothesis that HCMV activity, particularly UL88 expression, may influence tumor behavior and high-risk features in pediatric MB.

Studies suggest that HCMV can infect subependymal glia in the fourth ventricle, with reports of HCMV found in tumors in this area.^53^^,^^54^ This suggests that HCMV may be particularly associated with tumors originating from the rhombic lip subventricular zone (RLSVZ), primarily linked to non-WNT/non-SHH MBs of Groups ¾.^55^ Monocytes/macrophages could explain the presence of HCMV dissemination in tumors in this region.^3^^,^^12^^,^^13^ However, studies involving CSF have not succeeded in detecting CMV through PCR in confirmed infections.^47^^,^^48^ Further studies are needed to clarify the exact mechanisms of dissemination, and explained the origin of HCMV presence. Our findings indicate a potential involvement of CMV in the way MB tumors behave in pediatric patients, particularly in those exhibiting high-risk characteristics (M+, MYC/N-amplified). Furthermore, most cases with elevated expression are classified as non-WNT/non-SHH tumors, and none of the cases in our cohort showed TP53 mutations; therefore, we cannot determine HCMV expression in correlation to this risk feature. Larger cohort analyses are needed to confirm these correlations and better understand how HCMV influences MB. Further research is essential to determine how CMV may affect tumorigenesis through both inflammatory and genetic dysregulation in MB.

Recent studies involving glioblastoma patients have shown promising results using Valganciclovir (an antiviral targeting HCMV) as an adjuvant therapy for recurrent tumors.^56^^,^^57^ Murine MB models have demonstrated a significant reduction in tumor volume with a combination of Valganciclovir and Celecoxib therapy.^12^^,^^58^ Although HCMV vaccination in MB has been investigated in clinical trials, conclusive results have not yet been reported. Three clinical trials (NCT03615404, NCT05096481, NCT03299309) have examined vaccination against CMV in recurrent MB. Only one trial (NCT03615404) has been completed, showing promising results with a cohort of 11 patients.^59^^,^^60^ Given the aggressive nature of reoperation, radiotherapy, and CT in recurrent cases,^3^^,^^4^ antiviral treatment targeting HCMV may provide a potential prognostic and therapeutic approach for patients with recurrent MB who have HCMV expression in the future.

### Limitations

Our study has several limitations. It is a retrospective cohort with a small sample size (*n* = 45) and a favorable mortality rate of only 20% (*n* = 9), which restricted our OS analysis. Consequently, we evaluated EFS at 5 years and throughout the complete follow-up, considering only these two variables in the Cox analysis based on the total number of clinical or radiological adverse events (*n* = 21; including recurrence, progression, relapse, or death).^61^ This endpoint has been validated in studies of solid tumors in pediatric patients to address the limitations of OS endpoints.^36^^,^^37^ Additionally, MB is a diverse entity that is challenging to group, and each tumor requires an individualized approach. The risk stratification published by the SIOP-Europe/ERN PaedCan consensus considers risk stratums based on age groups and high-risk features [MYC/N amplified, LCA, R+, M+],^10^ which allows for a more reliable prognosis estimation in these patients. The survival rates in our cohort across risk groups were similar to those reported in the literature (5-year EFS of 89% for LR-MB, 73% for SR-MB, and 29% for HR-MB).^1^^,^^10^^,^^11^ However, HR-MB cases and HCMV-LA expression cases were both independently associated with significantly poor EFS.

Another limitation due to the retrospective nature is that we did not utilize frozen tissue samples or overnight incubation, which may explain the low HCMV-IE detection rate (*n* = 3). For our study, we conducted an optimized HCMV-IHC technique, with an antigen retrieval protocol, under standardized and reproducible conditions (60 min incubation in automated methods), with a published sensitivity of 84.2% in detecting protein in tumor tissue of FFPE.^45^ Our study aimed to investigate HCMV expression in archival tumor tissue (TMA/FFPE) utilizing optimized IHC. PCR and in situ hybridization (ISH) were not used due to their lower sensitivity for detecting HCMV genetic material in FFPE samples (29% and 38%, respectively) and the instability of HCMV genetic material in FFPE. We did not analyze frozen tissue specimens or cell cultures, which are recommended for PCR/ISH analysis. We did not therefore carry out PCR studies; IHC remains the most feasible technique for detecting not only the presence of HCMV but also the expression of antibodies within tumoral cells, thereby confirming HCMV’s presence in tumor tissue and identifying negative expression in regions without tumor.^45^ However, there is still no consensus on which studies are best for detecting HCMV in MB tumor tissue. Nonetheless, we base our study on previous publications on glioblastoma tissue analysis.^40^^,^^45^^,^^50^

To complement these findings, we analyzed WGS data from twenty pediatric MB tumors (21 datasets; EGAD00001000327; WNT *n* = 3, SHH *n* = 3, Group 3 *n* = 7, Group 4 *n* = 7) for HCMV (strain Merlin, NC_006273.2) expression. UL88 consistently exhibited the highest expression, suggesting a potential oncomodulatory role. Although most sequencing reads mapped to the human genome, the consistent viral transcripts support their biological relevance. Additionally, correlations between HCMV expression and inflammatory pathway genes (NF-κBp65, COX2, mPGES-1, phospho-STAT3) were evaluated in 251 pediatric MB cases via the R2 platform, confirming subgroup-specific patterns consistent with IHC results.

While this was beyond the scope of our retrospective study, we recognize it as an essential avenue for independent validation. Despite these limitations, our results demonstrate a significant association between HCMV-LA expression, high-risk MB features, and poor survival outcomes, with UL88 highest expression, particularly in Group 3 tumors. Further studies in larger pediatric cohorts—ideally prospective and including fresh-frozen tissue—are warranted to clarify HCMV’s role in MB pathogenesis and to validate its potential as a prognostic marker and therapeutic target.

## Conclusions

Our study demonstrates a significant association between HCMV-LA expression and poor survival outcomes in pediatric MB, particularly highlighting high HCMV-LA levels in tumors with high-risk features, including metastatic disease. This represents the first survival analysis in MB explicitly evaluating the relationship between HCMV expression in tumor tissue and clinical outcomes. Notably, WGS analysis revealed consistent HCMV gene expression, with UL88 exhibiting the highest expression, especially in high-risk Group 3 tumors, suggesting a potential oncomodulatory role. MB cases with elevated HCMV-LA—primarily within high-risk subgroups—demonstrated significantly worse survival, supporting HCMV as a potential prognostic biomarker. These results highlight the need for additional multicenter, prospective studies involving larger groups of pediatric patients. Ideally, these studies should include fresh-frozen tissue and detailed molecular analyses to confirm the prognostic significance of HCMV and assess its potential as a therapeutic target in pediatric MB.

## Supplementary Material

vdaf266_Supplementary_Data

## Data Availability

The datasets generated during and/or analyzed during the current study are available from the corresponding author on reasonable request.

## Previous Presentations

A portion of the abstract was presented at the AANS Annual Congress (Chicago, 2024), the ESPN Congress (London, 2025), and the 76th Annual Meeting of the German Society of Neurosurgery (DGNC, 2025).
