# Supplementary material for: High human cytomegalovirus antigen expression in pediatric medulloblastoma tissue is associated with poor event-free survival
Source: Neurooncol Adv. 2025 Dec 22;8(1):vdaf266. doi: 10.1093/noajnl/vdaf266 (PMC12932949; doi:10.1093/noajnl/vdaf266)
Supplement: vdaf266_Supplementary_Data [file vdaf266_supplementary_data.docx]

| **Variable** | **5-year event free survival (EFS)**  **(months)** | | | | | **EFS over the complete follow-up**  **(months)** | | | | | **Overall survival (months)** | | | | |
| --- | --- | --- | --- | --- | --- | --- | --- | --- | --- | --- | --- | --- | --- | --- | --- |
|  | **Events (*n/N*)** | **Mean** | **SD** | **Median** | **p-value (log-rank)** | **Events (n/N)** | **Mean** | **SD** | **Median** | **p-value (log-rank)** | **Events (n/N)** | **Mean** | **SD** | **Median** | **p-value (log-rank)** |
| Complete study cohort (n=45) | 21/45 | 84.81 | 12.79 | 52 |  | 21/45 | 90.23 | 10.95 | 94 |  | 9/45 | 129.93 | 11.43 | - | - |
| **Age** |  |  |  |  |  |  |  |  |  |  |  |  |  |  |  |
| ≤ 3 years old | 7/10 | 30.48 | 14.24 | 8 | 0.062 | 7/10 | 76.89 | 21.89 | 19 | 0.350 | 3/10 | 97.54 | 22.91 | - | 0.458 |
| > 3 years old | 14/35 | 96.28 | 14.50 | - |  | 14/35 | 94.97 | 13.51 | 84 |  | 6/35 | 130.63 | 14.07 | - |  |
| **Gender** |  |  |  |  |  |  |  |  |  |  |  |  |  |  |  |
| Male | 13/25 | 68.11 | 17.29 | 18 | 0.243 | 13/25 | 73.62 | 14.93 | 48 | 0.123 | 7/25 | 106.81 | 18.18 | 94 | 0.084 |
| Female | 8/20 | 89.29 | 15.88 | - |  | 8/20 | 103.67 | 13.06 | 147 |  | 2/20 | 133.47 | 9.73 | - |  |
| **Metastatic disease** |  |  |  |  |  |  |  |  |  |  |  |  |  |  |  |
| M0 | 7/27 | 123.77 | 14.30 | - | **<0.001*** | 7/27 | 126.31 | 12.35 | 129 | **<0.001*** | 4/27 | 142.45 | 11.88 | - |  |
| M1-M3 | 14/18 | 7.97 | 2.16 | 5 |  | 14/18 | 35.22 | 10.44 | 20 |  | 5/18 | 103.18 | 16.30 | - | 0.073 |
| **Preoperative hydrocephalus** |  |  |  |  |  |  |  |  |  |  |  |  |  |  |  |
| no | 3/16 | 119.32 | 14.77 | - |  | 3/16 | 117.70 | 15.02 | - | **0.018*** | 1/16 | 138.50 | 9.15 | - |  |
| yes | 18/29 | 58.34 | 14.63 | 13 | **0.006*** | 18/29 | 72.23 | 11.91 | 55 |  | 8/29 | 115.15 | 15.49 | - | 0.090 |
| **Fourth ventricle infiltration** |  |  |  |  |  |  |  |  |  |  |  |  |  |  |  |
| no | 10/28 | 98.57 | 17.71 | - | 0.257 | 10/28 | 106.49 | 14.70 | 147 | 0.079 | 4/28 | 142.87 | 11.92 | - |  |
| yes * | 11/17 | 56.34 | 13.20 | 18 |  | 11/17 | 66.28 | 13.23 | 64 |  | 5/17 | 89.76 | 13.83 | 94 | 0.235 |
| **Molecular group** |  |  |  |  |  |  |  |  |  |  |  |  |  |  |  |
| WNT-activated | 1/8 | 130.14 | 16.53 | - | 0.086 | 1/8 | 132.00 | 13.85 | - | 0.059 | 0/8 | - | - | - | 0.124 |
| SHH-activated and TP53-wt | 4/8 | 57.12 | 18.38 | 13 |  | 4/8 | 95.20 | 27.12 | 147 |  | 1/8 | 130.25 | 15.66 | - |  |
| Non-WNT/non-SHH | 16/29 | 64.93 | 15.65 | 18 |  | 16/29 | 72.64 | 12.87 | 55 |  | 8/29 | 109.44 | 16.90 | - |  |
| Group 3 | 7/12 | 51.54 | 17.46 | 18 | 0.179 | 7/12 | 76.95 | 17.87 | 94 | 0.126 | 4/12 | 89.65 | 16.16 | - | 0.223 |
| Group 4 | 9/17 | 65.60 | 20.94 | 18 |  | 9/17 | 68.14 | 17.40 | 48 |  | 4/17 | 108.21 | 26.04 | - |  |
| **MYC/N amplification** |  |  |  |  |  |  |  |  |  |  |  |  |  |  |  |
| no | 18/41 | 90.75 | 13.26 | 58 | **0.005*** | 18/41 | 96.59 | 11.22 | 94 | **<0.001*** | 7/41 | 135.75 | 11.23 | - | **0.013*** |
| yes | 3 /4 | 5.50 | 3.36 | - |  | 3 /4 | 13.66 | 3.48 | 13 |  | 2/4 | 13.66 | 2.84 | 13 |  |
| **Extend of resection (EOR)** |  |  |  |  |  |  |  |  |  |  |  |  |  |  |  |
| RTV no or < 1.5 cm^2^ | 15/36 | 97.25 | 13.75 | - | **0.001*** | 15/36 | 100.49 | 12.06 | 114 | **0.015*** | 6/36 | 136.63 | 11.77 | - | 0.054 |
| RTV ≥ 1.5 cm^2^ | 6/9 | 5.75 | 2.00 | 5 |  | 6/9 | 42.86 | 24.54 | 19 |  | 3/9 | 89.48 | 26.01 | - |  |
| **MB Risk groups** |  |  |  |  |  |  |  |  |  |  |  |  |  |  |  |
| LR-MB | 1/9 | 134.11 | 13.09 | - |  | 1/9 | 135.20 | 11.44 | - |  | 0/9 | - | - | - |  |
| SR-MB | 4/15 | 112.11 | 22.96 | - |  | 4/15 | 119.04 | 18.90 | 129 |  | 3/15 | - | - | - |  |
| HR-MB | 16/21 | 19.19 | 6.68 | 7 | **<0.001*** | 16/21 | 47.43 | 11.22 | 38 | **<0.001*** | 6/21 | - | - | - | 0.096 |
| **grouped MB risk groups** |  |  |  |  |  |  |  |  |  |  |  |  |  |  |  |
| non-HR MB | 5/24 | 131.90 | 14.41 | - |  | 5/24 | **132.42** | **13.37** | **-** |  | 3/24 | 145.61 | 12.29 | - | 0.080 |
| HR-MB | 16/21 | 19.19 | 6.68 | 7 | **<0.001*** | 16/21 | **47.43** | **11.22** | **38** | **<0.001*** | 6/21 | 95.05 | 17.83 | - |  |
| **HCMV-LA expression levels** |  |  |  |  |  |  |  |  |  |  |  |  |  |  |  |
| Low expression | 6/21 | 120.42 | 16.33 | - | **0.003*** | 6/21 | 129.08 | 13.07 | 147 | **<0.001*** | 4/21 | 138.47 | 13.43 | - | 0.368 |
| High expression | 15/24 | 25.77 | 6.64 | 8 |  | 15/24 | 48.25 | 9.41 | 39 |  | 5/24 | 88.94 | 9.82 | - |  |

**Supplementary material.**

**Table 1.- Univariate Survival analysis considering 5-year event-free survival (EFS), EFS over the complete follow-up period, and Overall survival in the study cohort.**

A total of 21/45 adverse events (47%) (including recurrence, progression, relapse, or death) were observed in the cohort study (n=45). The 5-year event-free survival (EFS) analysis utilized the date of the first clinical adverse event (recurrence, progression, relapse, or death). For EFS throughout the complete follow-up period, the date of the last clinical adverse event was considered in cases with a single or multiple adverse events. Overall survival (OS) was calculated using death (n=9) as the sole event. The survival data were measured in months. Events (n/N) = Events Per Variable. * non-WNT/non-SHH with infiltration in the fourth ventricle. ** LR-MB = low-risk MB, SR-MB = standard risk MB, and HR-MB = high-risk MB (according to SIOP-Europe/ ERN PaedCan). ***HCMV-LA expression levels are classified as low (0 or less than 25% positive cells) and high (≥ 25% positive tumor cells).

The p-value was calculated using log-rank, with p<0.05 considered statistically significant.

**Table 2. Cox analysis for 5-year event-free survival (EFS) and EFS in the complete follow-up.**

**Hazard ratio (HR) less than 1 indicates a better outcome; >1 indicates a worse outcome.**

| **Variable** | **Events (*n/N*)** | **5-year event-free survival (EFS)** | | | | **Events (n/N)** | **EFS in the complete follow-up** | | | |
| --- | --- | --- | --- | --- | --- | --- | --- | --- | --- | --- |
|  |  | **HR** | **95% CI (lower)** | **95% CI (upper)** | **p-value** |  | **HR** | **95% CI (lower)** | **95% CI (upper)** | **p-value** |
| **Age** |  |  |  |  |  |  |  |  |  |  |
| ≤ 3 years old | 7/10 | 3.521 | 1.313 | 9.441 | **0.012*** | 7/10 | 1.687 | 0.524 | 5.429 | 0.381 |
| > 3 years old | 14/35 | 1 |  |  |  | 14/35 | 1 |  |  |  |
| **Gender** |  |  |  |  |  |  |  |  |  |  |
| Male | 13/25 | 1 |  |  |  | 13/25 | 1 |  |  |  |
| Female | 8/20 | 0.876 | 0.355 | 2.161 | 0.774 | 8/20 | 0.340 | 0.113 | 1.028 | 0.056 |
| **Risk groups** |  |  |  |  |  |  |  |  |  |  |
| Non-HR MB | 5/24 | 1 |  |  |  | 5/24 | 1 |  |  |  |
| HR MB | 16/21 | 6.594 | 2.046 | 21.257 | **0.002*** | 16/21 | 4.197 | 1.239 | 14.218 | **0.021*** |
| **HCMV-LA** |  |  |  |  |  |  |  |  |  |  |
| Low expression | 6/21 | 1 |  |  |  | 6/21 | 1 |  |  |  |
| High expression | 15/24 | 2.555 | 0.818 | 7.974 | 0.106 | 15/24 | 4.334 | 1.184 | 15.857 | **0.027*** |
| Omnibus χ² = 27.765, df = 4, *p*<0.001 | | | | | | Omnibus χ² = 24.789, df = 4, *p*<0.001 | | | | |

Events (n/N) = Events Per Variable. Non-HR MB= including low-risk and standard risk MB, and HR-MB = high-risk MB (according to SIOP-Europe/ ERN PaedCan). HCMV-LA expression levels are classified as low (0 or less than 25% positive cells) and high (≥ 25% positive tumor cells). The 5-year event-free survival (EFS) analysis used the initial clinical adverse event date (recurrence, progression, relapse, or death). For EFS during the entire follow-up period, the date of the last clinical adverse event was considered for cases with either a single or multiple adverse events.

The p-value p<0.05 was considered statistically significant.

***
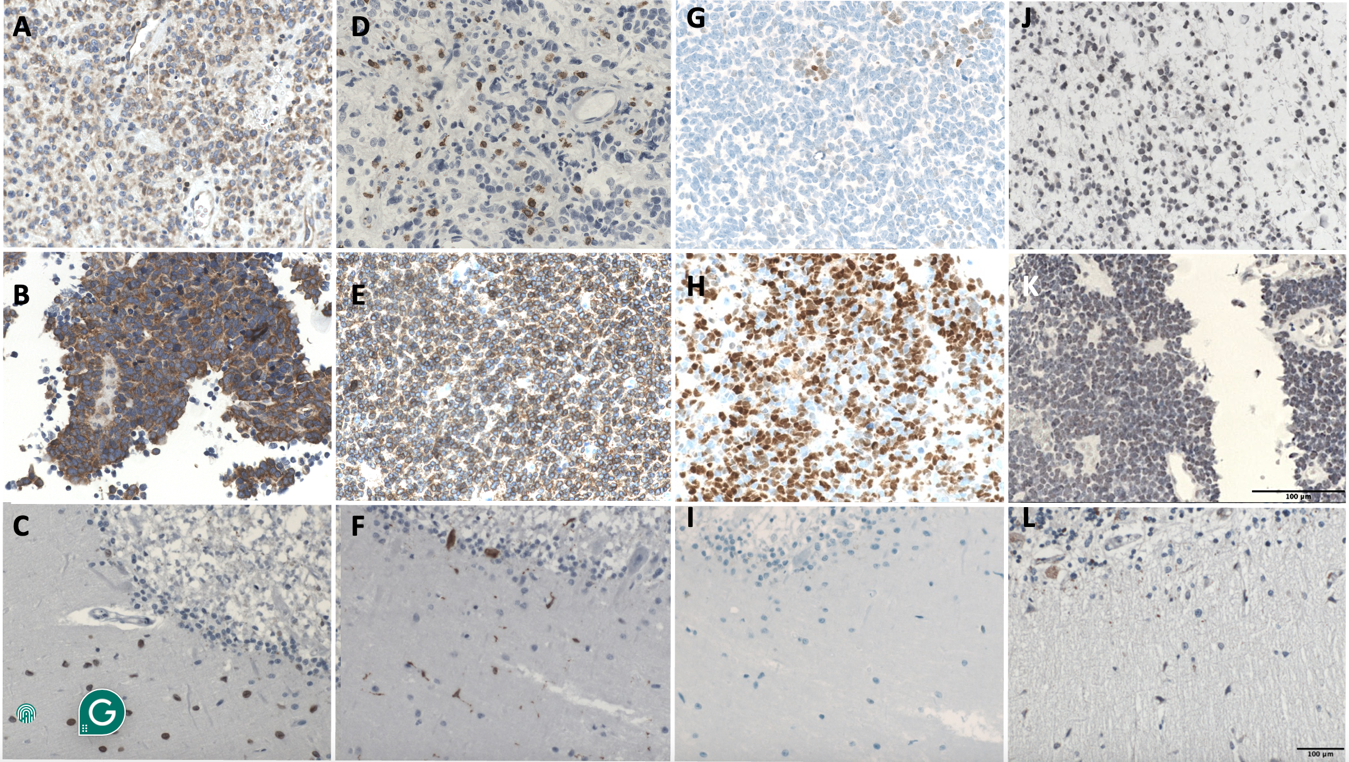
***

**Figure 1.** *Immunohistochemistry staining for inflammatory markers.* **NF-κB p65** staining shows low expression (zero or <25% positive tumor cells) (**A**), high expression (≥25% positive tumor cells) (**B**), and the normal cerebellum exhibiting diffuse nuclear reactivity (**C**). **COX-2** staining reveals low expression (zero or <25% in **D**), high expression (≥25% in **E**), and the normal cerebellum demonstrating diffuse reactivity in glial cells (**E**). **Phospho-STAT3** immunostaining indicates low expression (zero or <25%) (**G**), high expression (≥25%) (**H**), and a normal cerebellum with no immunoreactivity (**J**). **mPEGS-1** staining shows low expression (zero or <25%) (**K**), high expression (≥25%) (**H**), and a normal cerebellum exhibiting no reactivity (**L**).


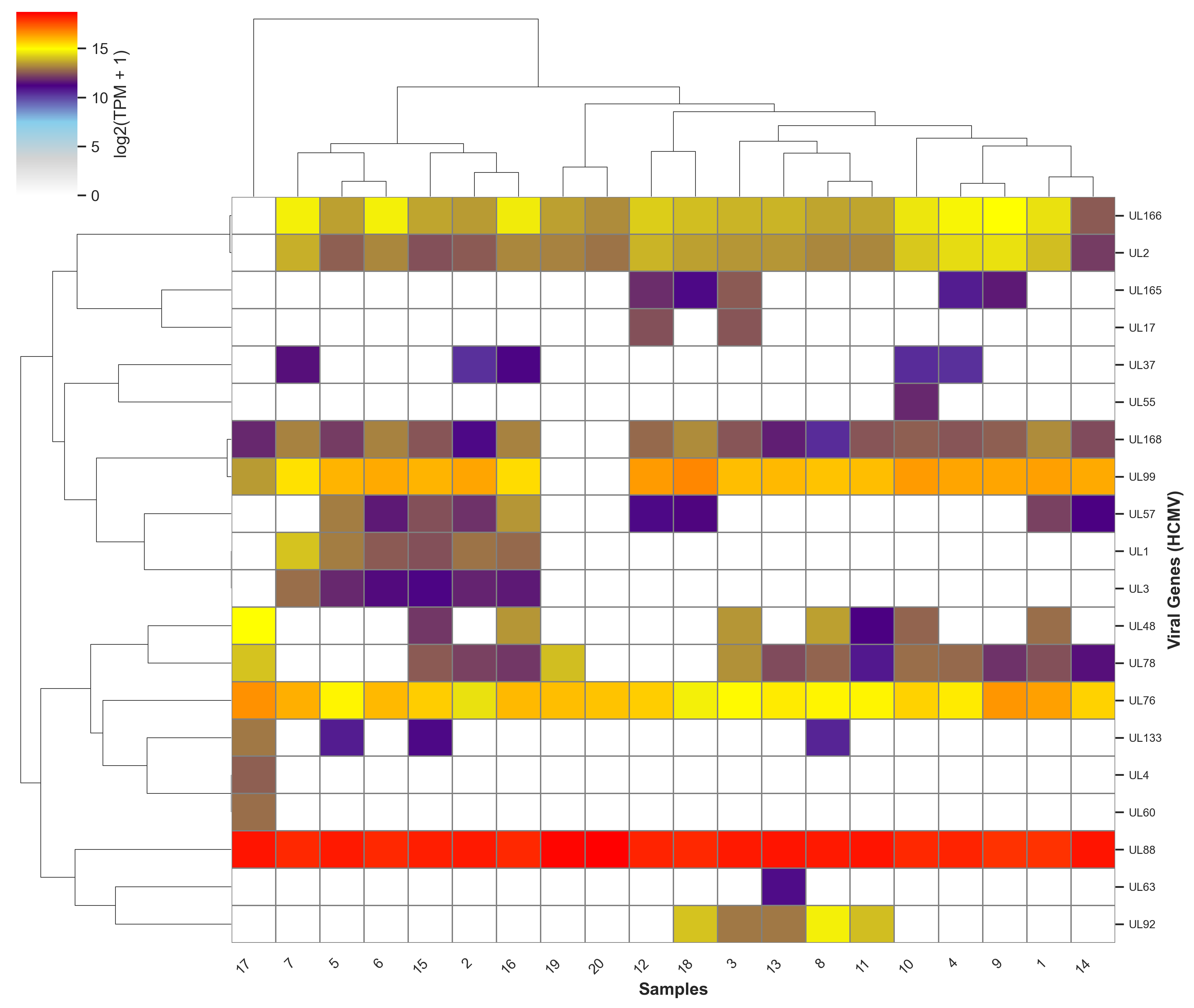


**Figure 2. Heatmap of HCMV gene expression across medulloblastoma samples.** Heatmap showing the expression levels (log₂[TPM + 1]) of selected human cytomegalovirus (HCMV) genes across 20 pediatric medulloblastoma samples. Hierarchical clustering of samples and genes reveals consistent expression of UL88, UL60, UL99, and UL76. UL88 exhibited the strongest expression signal (log₂[TPM + 1] ≈ 14–15, equivalent to ~16,000–33,000 TPM), followed by UL60 and UL99. This pattern highlights UL88 as the most abundantly expressed HCMV gene across medulloblastoma samples.


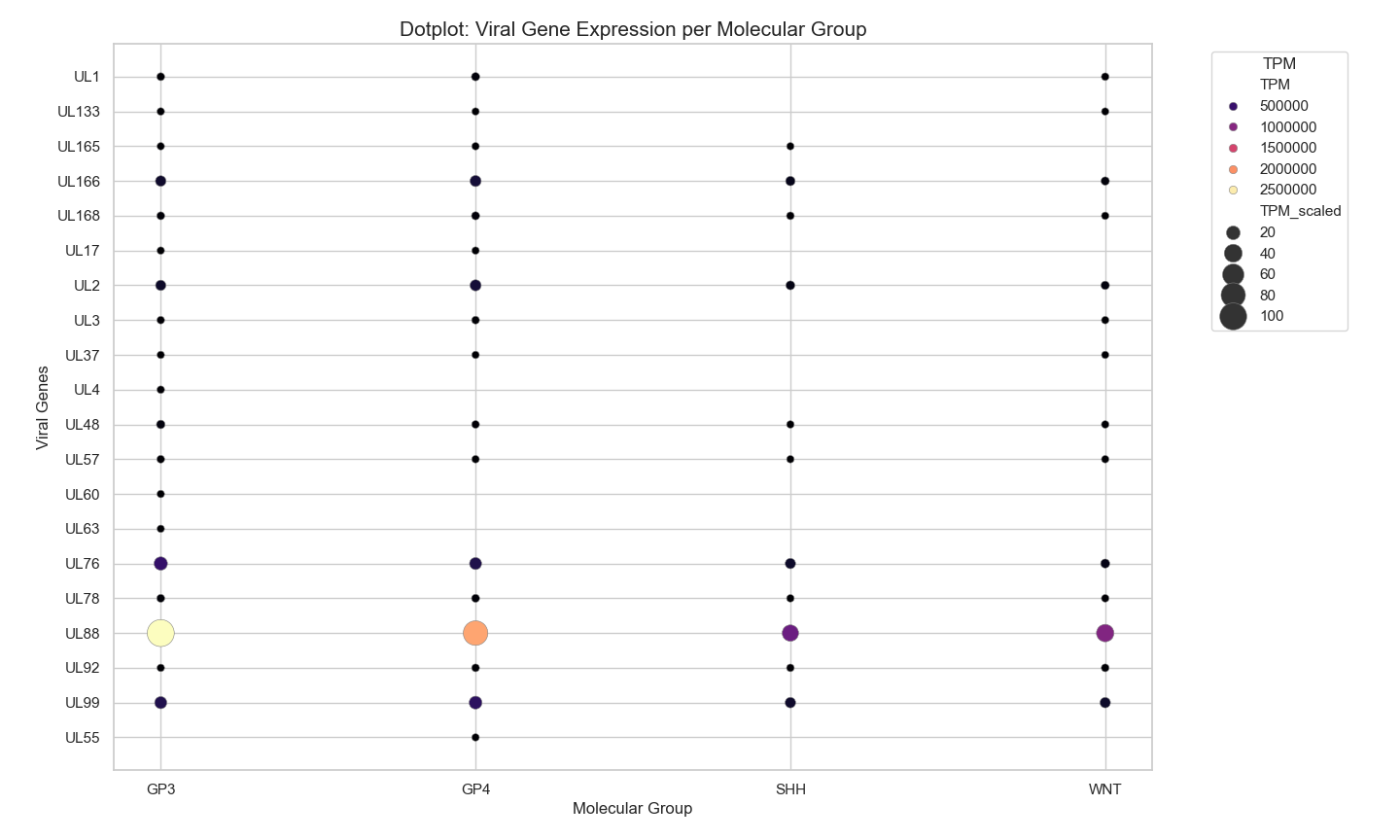


**Figure 3. Dot plot of HCMV gene expression across medulloblastoma molecular subgroups.** The dot plot illustrates viral gene expression in the WNT, SHH, Group 3 (GP3), and Group 4 (GP4) subgroups. Dot color and size represent expression levels (TPM). UL88 exhibits the strongest and most consistent expression, particularly in Group 3 (TPM ≈ 2,500,000, yellow dot) and Group 4 (TPM ≈ 2,000,000, orange dot), indicating subgroup-specific viral activity and a potential oncomodulatory role.


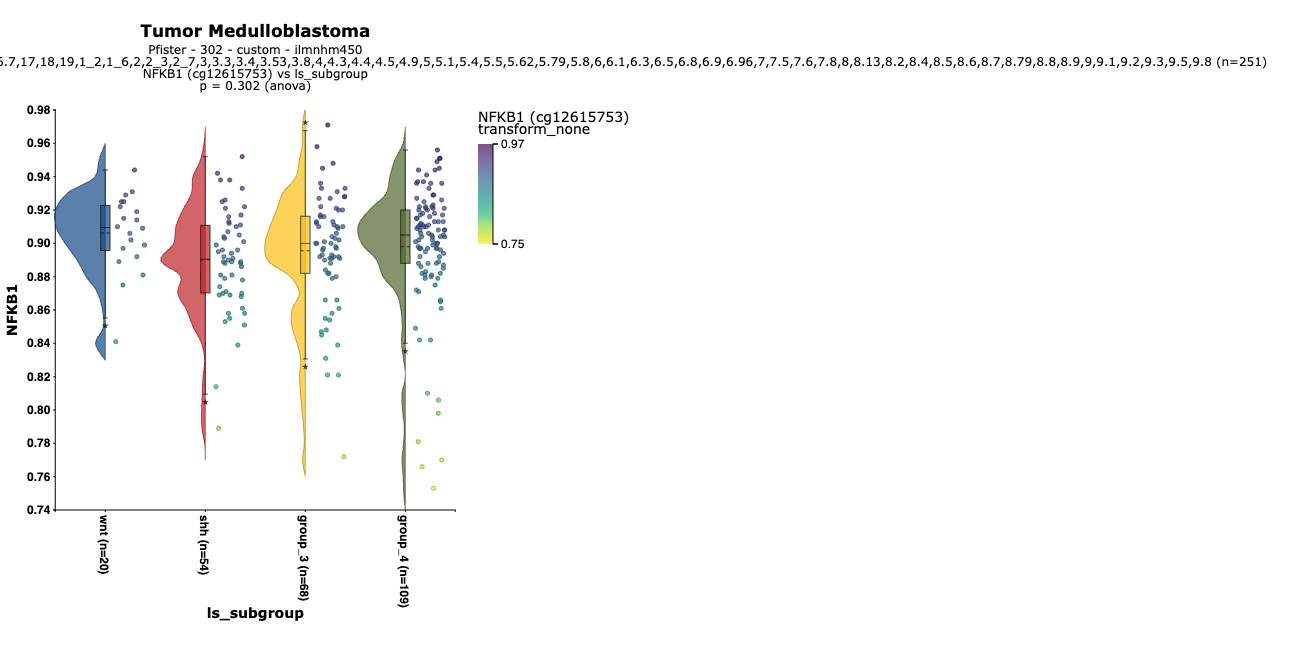

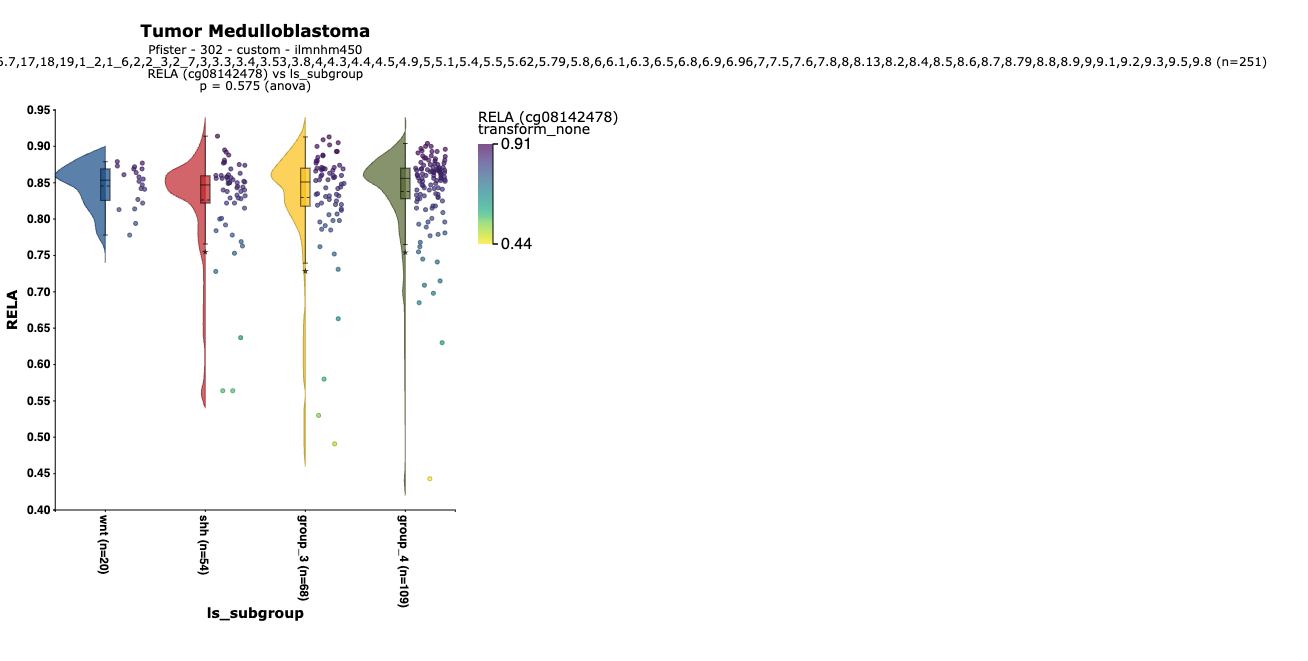

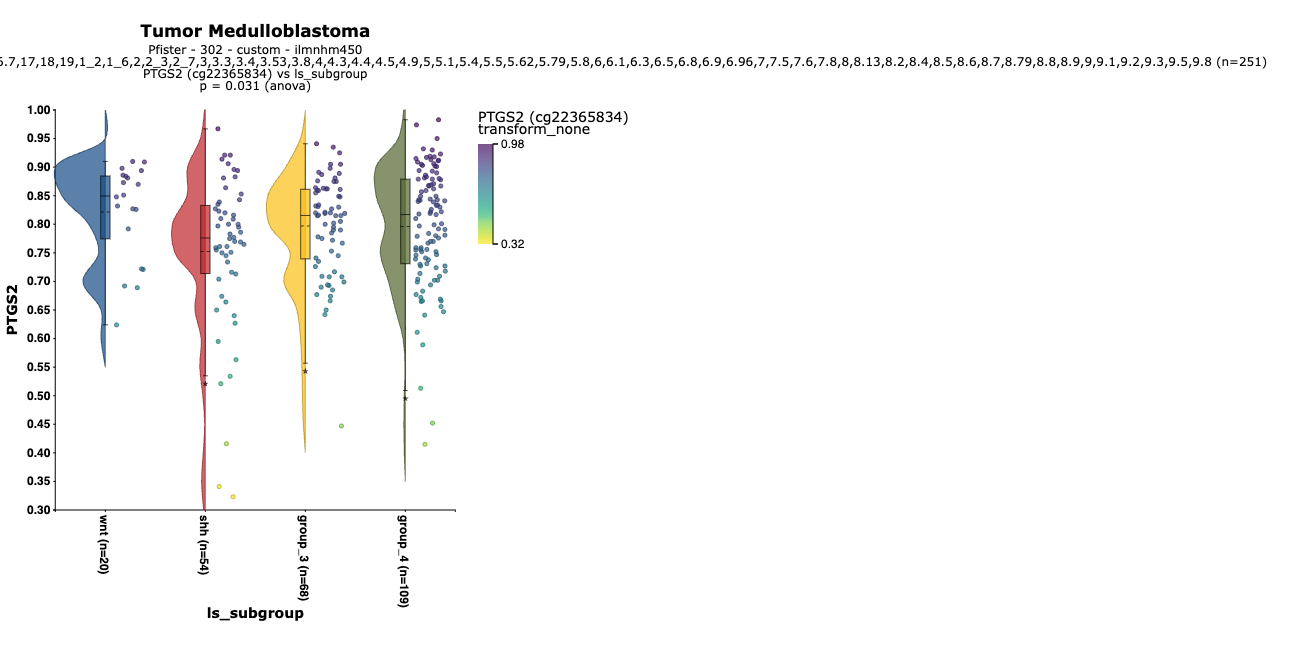


**A B**

**C D**

**E**

(n=251)


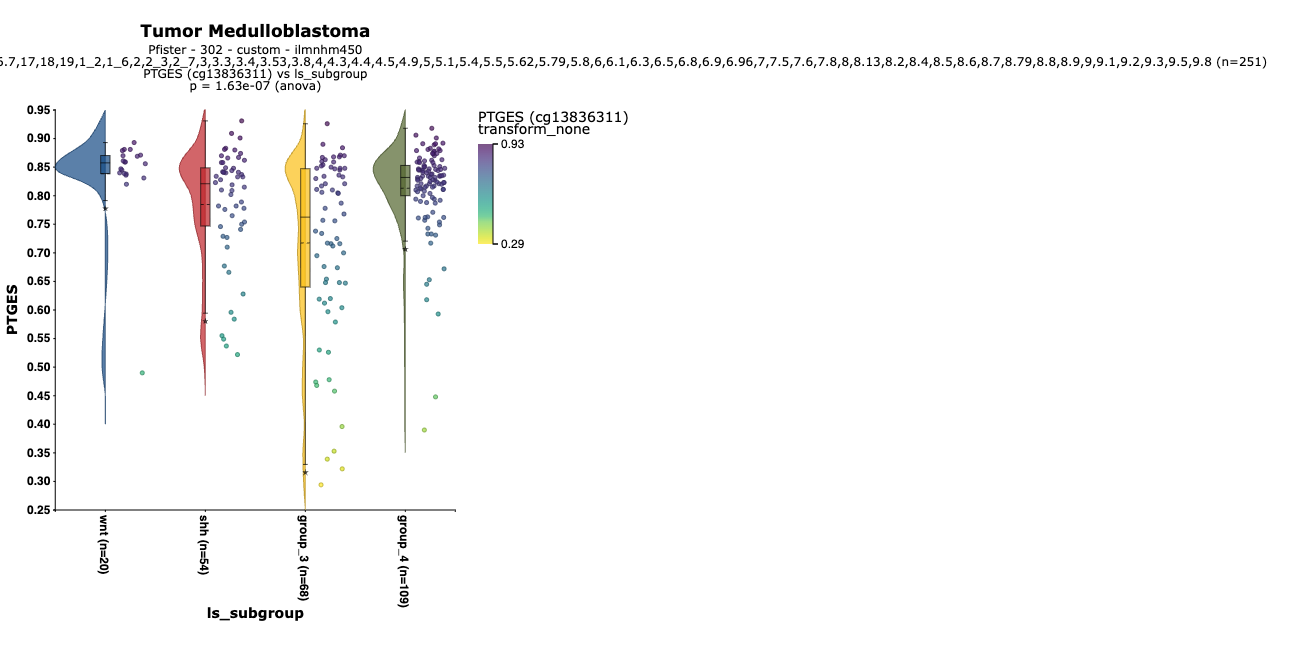

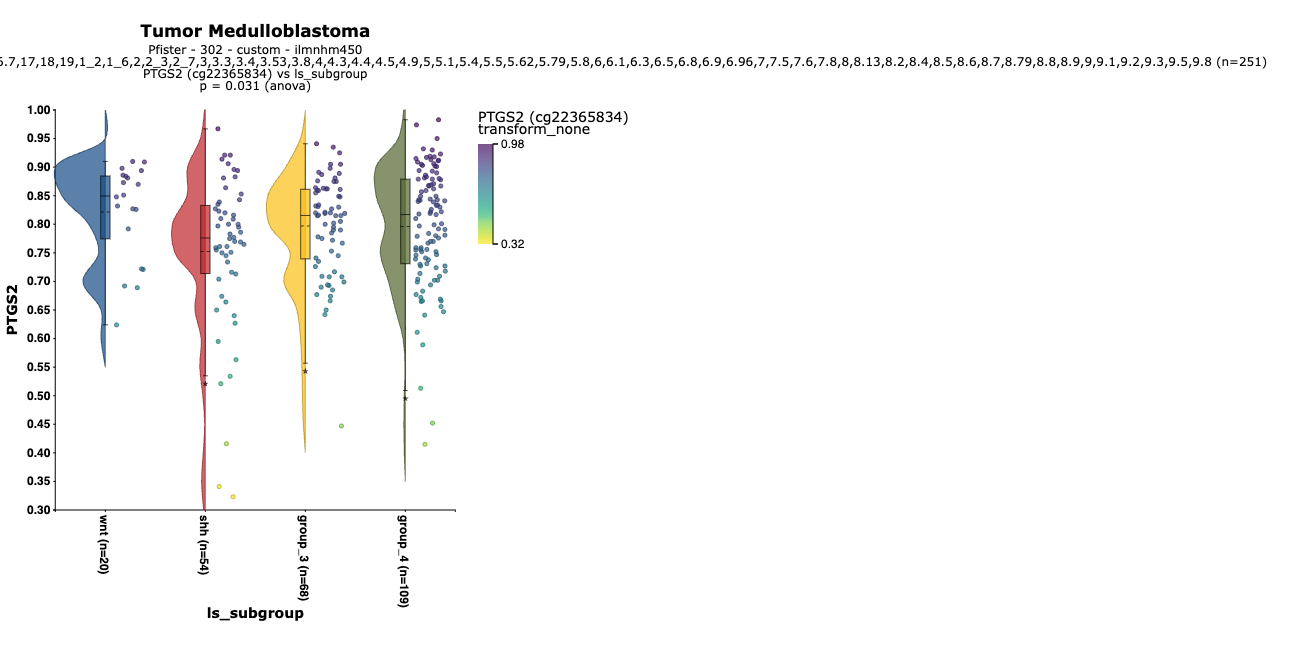


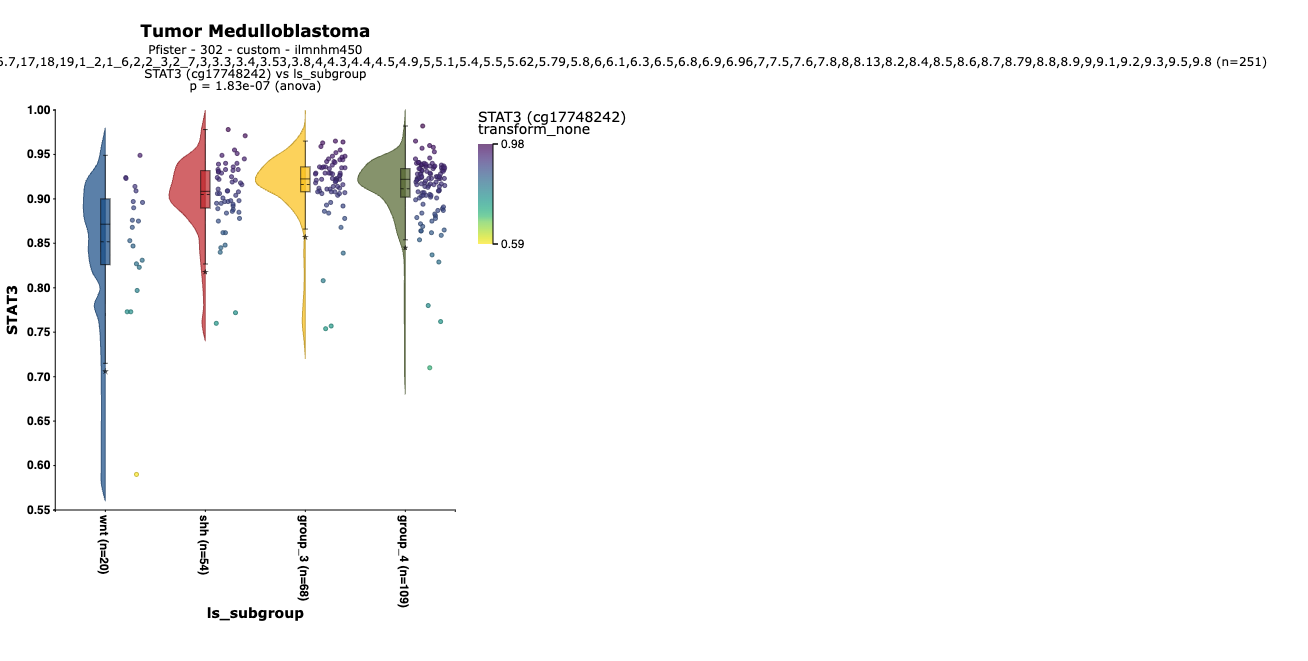


**Figure 4. Raincloud plots of inflammatory pathway gene expression in pediatric medulloblastoma samples analyzed using the R2 platform (Medulloblastoma Tumor – Pfister – 302 – custom – ILMN450 dataset), which includes DNA methylation and expression data from 302 samples.** Only pediatric cases (n = 251, age ≤19 years) were included. Expression of NF-κBp65 (A–RELA, B–NFKB1), COX2 (C–PTGS2), mPGES-1 (D–PTGES), and STAT3 (E–phospho-STAT3) is shown across molecular subgroups: WNT (blue), SHH (red), Group 3 (yellow), and Group 4 (green). C–PTGS2 expression was significantly higher in WNT tumors (median = 0.85, Q25–Q75 = 0.77–0.88) compared with other subgroups (ANOVA: F = 4.015, p = 0.031). Similarly, D–PTGES expression was elevated in WNT tumors (median = 0.86, Q25–Q75 = 0.84–0.89; ANOVA: F = 12.276, p = 1.63×10⁻⁷). E–STAT3 expression was significantly higher in Group 3 (median = 0.92, Q25–Q75 = 0.91–0.94) and Group 4 tumors (median = 0.92, Q25–Q75 = 0.90–0.93; ANOVA: F = 12.187, p = 1.83×10⁻⁷). In contrast, A–RELA and B–NFKB1 did not show significant differences across subgroups (RELA: F = 0.664, p = 0.575; NFKB1: F = 1.223, p = 0.302). These results are consistent with immunohistochemistry findings, indicating that activation of the COX2/PGE2 inflammatory pathway is characteristic of WNT medulloblastomas, whereas NF-κB signaling does not exhibit subgroup-specific variation.
